# Supplementary material for: Jointly benchmarking small and structural variant calls with vcfdist
Source: Genome Biol. 2024 Oct 2;25:253. doi: 10.1186/s13059-024-03394-5 (PMC11446017; doi:10.1186/s13059-024-03394-5)
Supplement: Supplementary file 1 — Additional file 1. Contains the following tables and figures: Table S1: The origins of each phased whole genome sequencing dataset used in this manuscript. Figure S1: The design space for affine-gap alignment and variant representations. Figure S2: An example where joint evaluation of small and structural variants changes benchmarking results. Figure S3: Switch and flip error confusion matrices for vcfdist and WhatsHap. Figure S4: Full haplotype sequences for the truth and query VCFs for a portion of the HLA-DQB1 gene. Figure S5: A comparison of SNP, INDEL, and SV variant calling evaluation by vcfdist, vcfeval, and Truvari. Figure S6: Examples of the seven variant categories in Table 4 that were evaluated differently between tools. Table S2: Comparing the efficiency and accuracy of vcfdist when using different clustering approaches. Figure S7: Comparing detected and actual variant dependencies when using different clustering algorithms. Figure S8: An example of different variant clustering methods. [file 13059_2024_3394_MOESM1_ESM.pdf]

Additional File 1

Datasets

	Q100-dipcall	hifiasm-dipcall	Q100-PAV	hifiasm-GIAB-TR
**Consortium(s)**	T2T, GIAB, HPRC	HPRC	NIST	GIAB
**Assembler**	verkko	hifiasm	verkko	hifiasm
**Assembly Method**	trio-based	trio-based	trio-based	trio-based
**Assembly**	T2T-HG002-Q100v0.9 polished	HPRC scaffold	T2T-HG002-Q100v0.9 polished	HPRC scaffold
**Variant Caller**	minimap2, dipcall	minimap2, dipcall	PAV	minimap2, paftools

Table S1: The origins of each phased whole genome sequencing dataset used in this manuscript.

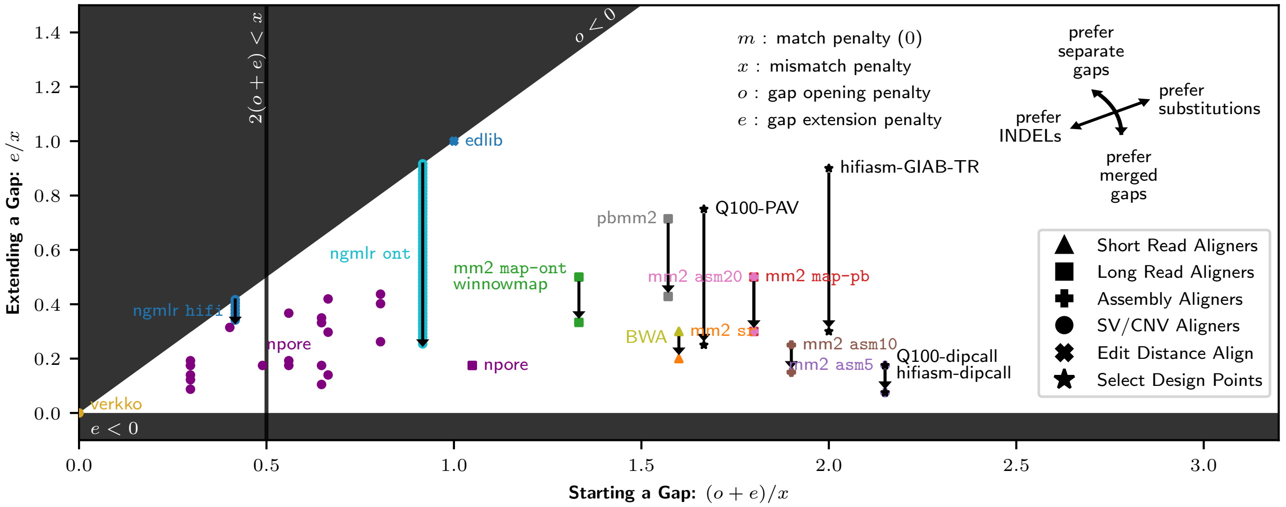

Fig. S1: The design space for affine-gap alignment and variant representation with match, mismatch, gap opening, and gap extension penalties m, x, o, and e. All parameters have been normalized so that $m = 0$, and the penalties for starting $(o + e)$ and extending (e) a gap are plotted relative to substitutions (x). This plot includes the variant representations used in all four datasets, along with short-read, long-read, assembly, edit distance, copy number variant, and structural variant aligners for comparison. Each aligner is plotted in a unique color, except for when multiple aligners use identical parameters. For dual affine gap aligners, two points are plotted with an arrow indicating the transition to a lower extension penalty e_2. NGMLR uses a logarithmic gap penalty, and so there is a continuous lowering of e. verkko is plotted at $(0, 0)$ because it uses tandem repeat compression. nPoRe uses different gap penalties for simple tandem repeats (STRs) based on their measured likelihoods, resulting in many plotted points.

Joint Benchmarking Example

SMALL

CONTIG	POS	REF	ALT	FORMAT	TRUTH	QUERY
chr1	996555	T	C	GT:BD:BC	1|1:FN:0.000	.:.:.
chr1	996559	G	A	GT:BD:BC	.:.:.	1|1:FP:0.000
chr1	996596	A	AGGACCCCCCGCTGGAGGGG	GT:BD:BC	.:.:.	1|1:FP:0.000
chr1	996674	G	T	GT:BD:BC	1|1:TP:1.000	1|1:TP:1.000
chr1	996691	G	A	GT:BD:BC	1|1:FN:0.000	.:.:.
chr1	996707	T	G	GT:BD:BC	.:.:.	1|1:FP:0.000
chr1	996722	G	A	GT:BD:BC	1|1:TP:1.000	1|1:TP:1.000
chr1	996728	A	AG	GT:BD:BC	.:.:.	1|1:FP:0.000
chr1	996731	A	C	GT:BD:BC	1|1:TP:1.000	1|1:TP:1.000

SV

CONTIG	POS	REF	ALT	FORMAT	TRUTH	QUERY
chr1	996282	G	GGGGGCACCCCACATCTGGGGCCACAGGATGCAG<br>GGTGGGGAGGGCAGAAAGGCCCCCCCGCGGGAA	GT:BD:BC	1|1:TP:0.867	.:.:.
chr1	996348	A	AGGGGCACCCCACATCTGGGGCCACAGGATGCAG<br>GGTGGGGAGGGCAGAAAGGCCCCCCCGCGGGAAG<br>GGGCACCCCACATCTGGGGCCACAGGATGCAGGG<br>TGGGGAGGGCAGAAAGGACCCCCCGCTGGAGGGG<br>GCACCTCACGTCTGGGGCCACAGGATGCAGGGTG<br>GGGAGGACAGAAAGGACCCCCCGCTGGAG	GT:BD:BC	.:.:.	1|1:TP:0.867
chr1	996728	A	AGGACCCCCCGCTGGAGGGGGGACCCCCCGCTGG<br>AGGGGGCACCCCACATCTGGGGCCACAGGATGCA<br>GGGTGGGGAGGGCAGAAAGGACCCCCCGCTGGAG<br>GGGGCACCTCACGTCTGGGGCCACAGGAGGCAGG<br>GTGGGGAGGACAGAAAG	GT:BD:BC	1|1:TP:0.867	.:.:.

ALL

CONTIG	POS	REF	ALT	FORMAT	TRUTH	QUERY
chr1	996282	G	GGGGGCACCCCACATCTGGGGCCACAGGATGCAG<br>GGTGGGGAGGGCAGAAAGGCCCCCCCGCGGGAA	GT:BD:BC	1|1:TP:1.000	.:.:.
chr1	996348	A	AGGGGCACCCCACATCTGGGGCCACAGGATGCAG<br>GGTGGGGAGGGCAGAAAGGCCCCCCCGCGGGAAG<br>GGGCACCCCACATCTGGGGCCACAGGATGCAGGG<br>TGGGGAGGGCAGAAAGGACCCCCCGCTGGAGGGG<br>GCACCTCACGTCTGGGGCCACAGGATGCAGGGTG<br>GGGAGGACAGAAAGGACCCCCCGCTGGAG	GT:BD:BC	.:.:.	1|1:TP:1.000
chr1	996350	A	G	GT:BD:BC	1|1:TP:1.000	1|1:TP:1.000
chr1	996423	C	T	GT:BD:BC	1|1:TP:1.000	.:.:.
chr1	996555	T	C	GT:BD:BC	1|1:TP:1.000	.:.:.
chr1	996559	G	A	GT:BD:BC	.:.:.	1|1:TP:1.000
chr1	996596	A	AGGACCCCCCGCTGGAGGGG	GT:BD:BC	.:.:.	1|1:TP:1.000
chr1	996674	G	T	GT:BD:BC	1|1:TP:1.000	1|1:TP:1.000
chr1	996691	G	A	GT:BD:BC	1|1:TP:1.000	.:.:.
chr1	996707	T	G	GT:BD:BC	.:.:.	1|1:TP:1.000
chr1	996722	G	A	GT:BD:BC	1|1:TP:1.000	1|1:TP:1.000
chr1	996728	A	AG	GT:BD:BC	.:.:.	1|1:TP:1.000
chr1	996728	A	AGGACCCCCCGCTGGAGGGGGGACCCCCCGCTGG<br>AGGGGGCACCCCACATCTGGGGCCACAGGATGCA<br>GGGTGGGGAGGGCAGAAAGGACCCCCCGCTGGAG<br>GGGGCACCTCACGTCTGGGGCCACAGGAGGCAGG<br>GTGGGGAGGACAGAAAG	GT:BD:BC	1|1:TP:1.000	.:.:.
chr1	996731	A	C	GT:BD:BC	1|1:TP:1.000	1|1:TP:1.000

Fig. S2: An example (from the Q100-PAV dataset) where joint evaluation of small and structural variants changes the benchmarking results from 4 true positives (`TP`), 4 false positives (`FP`), and 2 false negatives (`FN`) to 9 true positives. Each variant call file (VCF) entry reports the variant contig (`CONTIG`), position (`POS`), reference allele (`REF`), alternate allele (`ALT`), and truth and query information: genotypes (`GT`), benchmarking decision (`BD`), and benchmarking credit (`BC`).

Phasing Analysis

In Additional File 1: Fig. S3b, vcfdist classifies a cluster's flip error status as `NONE` when the original haplotypes match exactly, and as `FLIP` when both haplotypes match exactly when the phasing of all variants is flipped. Because the full haplotypes match exactly, the ground truth is known and vcfdist is correct. When neither phasing results in an exact match, the ground truth is labelled `UNKNOWN` but vcfdist still makes a classification (`NONE`/`FLIP`) based on how much closer the edit distance between the truth and query haplotypes is when all variant phasings are flipped. Because the hifiasm-dipcall dataset uses the same variant representation as the Q100-dipcall ground truth, there

are few WhatsHap false positive flip errors for this VCF. Once the query VCF variant representation differs from the ground truth, however, the Q100-PAV and hifiasm-GIAB-TR datasets show a large number of WhatsHap false positive flip errors in Additional File 1: Fig. S3b.

Because vcfdist enforces local phasing of variants within a cluster, it may miss single-variant flip errors when the phasing of one of several variants in a cluster is incorrect. In this case, the ground truth would be labelled `UNKNOWN` because neither phasing would result in the truth and query haplotypes matching exactly. A manual investigation of a random subset of these cases with unknown ground truth in Additional File 1: Fig. S3c shows that in most of these cases, no flip error has occurred. This shows that although vcfdist reports far fewer flip errors in total than WhatsHap, few of these are false negatives. The majority of cases with unknown ground truth are likely also WhatsHap false positives.

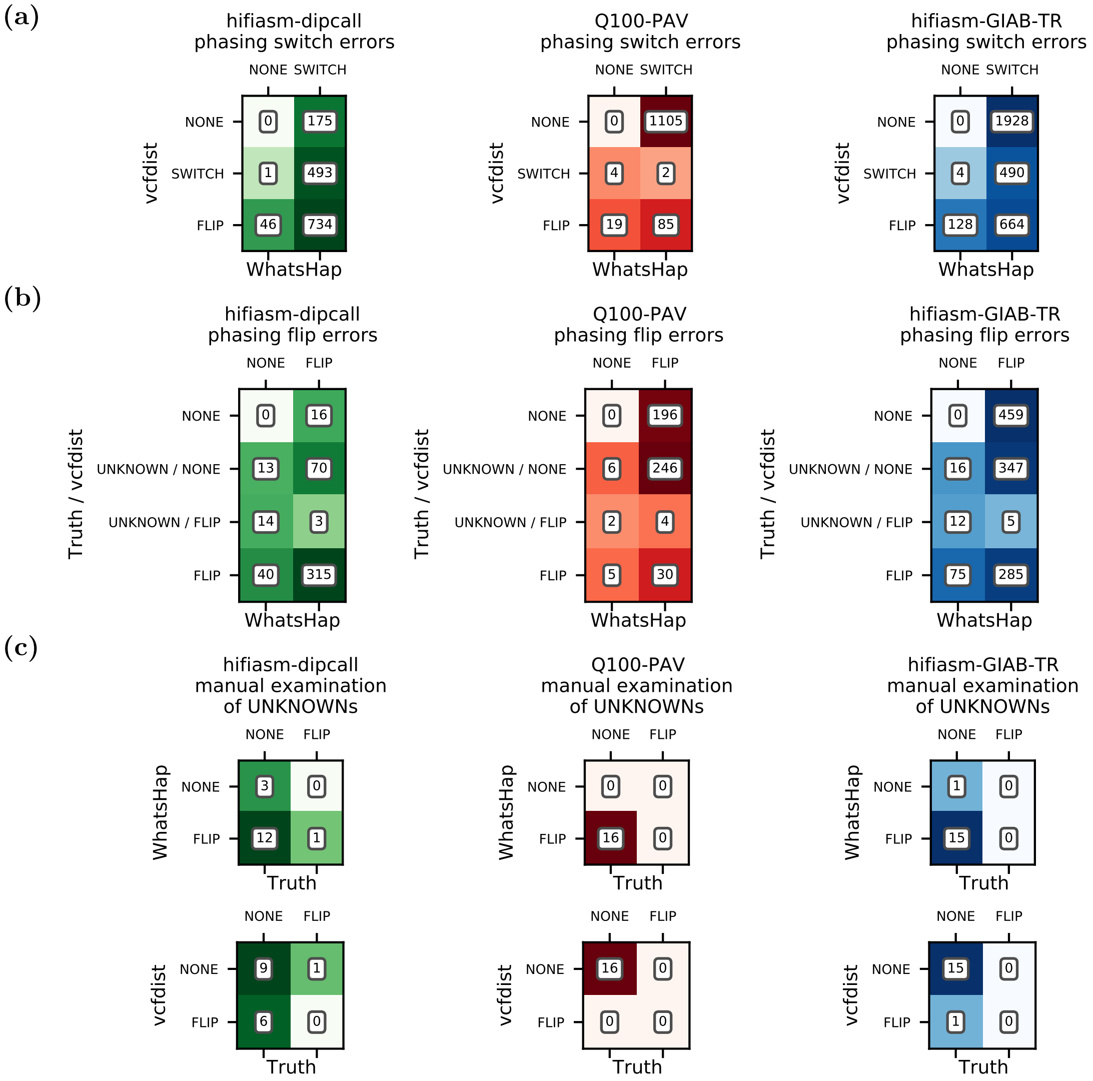

Fig. S3: **(a)** Switch error confusion matrices for vcfdist and WhatsHap for all three whole genome sequencing datasets, evaluated on the GIAB-Q100 BED. Note that WhatsHap considers flips errors to be equivalent to two consecutive switch errors, whereas vcfdist counts each separately. **(b)** Flip error confusion matrices for vcfdist and WhatsHap. Note that cluster ground truths are only labelled as `NONE` or `FLIP` when absolutely certain, i.e. if both haplotypes with all variants using either the original or flipped phasing match *exactly*. As a result, many WhatsHap false positive flip calls can be identified. An example is shown in Figure 4. **(c)** For `UNKNOWN` clusters, we manually examined a random subset of 16 clusters from each VCF to determine the ground truth and plot confusion matrices for WhatsHap and vcfdist.

HLA-DQB1 Complex Variant Example

Q100-dipcall haplotype 0

Q100-dipcall hap 0, hifiasm-dipcall hap 0

```
CTGCCTGTCCCCCTGCTCTGCCCTAGGTCCCTGCCCCTCCGATGCACCAGCCCCCAGCAC
CCCCACCGCCTCCTCCTGTCAGCCGGGGTGGAACGAACAAGGCTCAGGTTCCAGAGGCCG
CGCCCCCTTCGCCCCTCCTGGCGCAGAGACTCTGGGCCCCTGCCAAGGATGGCCTCGCA
GACGGGCGACGACGCTCACCTCGCCGCTGCAAGGTCGTGCGGAGCTCCAACTGCCGCCGC
CTCCTTTCCCCTGGGGTGGAATGAACTGGGCTCAGATTTCAGAGACCTCGCCCCCATCGC
CCCTCCCGGCACAGAAACTCGGGGTCTCGGCCAAGGGTGGGCCTCACGGAGGGGCGACGA
CGCTCACCTCTCCTCTGCAAGATCCCGCGGAACGCCACCTCGTAGTTGTGTCTGCACACC
GTGTCCAACTCCGCCCGGGTCCCTTCCAGGACTTCCTTCTGGCTGTTCC
```

Q100-PAV hap 0, hifiasm-GIAB-TR hap 1

```
CTGCCTGTCCCCCTGCTCTGCCCTAGGTCCCTGCCCCTCCGATGCACCAGCCCCCAGCAC
CCCCACCGCCTCCTCCTGTCAGCCGGGGTGGAACGAACAAGGCTCAGGTTCCAGAGGCCG
CGCCCCCTTCGCCCCTCCTGGCGCAGAGACTCTGGGCCCCTGCCAAGGATGGCCTCGCA
GACGGGCGACGACGCTCACCTCGCCGCTGCAAGGTCGTGCGGAGCTCCAACTGGTAGTTG
TGTCTGCACACCGTGTCCAACTCCGCCCGGGTCCCTTCCAGGACTTCCTTCTGGCTGTTC
C
```

Q100-dipcall haplotype 1

Q100-dipcall hap 1, hifiasm-dipcall hap 1, Q100-PAV hap 0

```
CTGCTTGTCTCCCTGCTCTGCCCTAGGTCCCCGCCCATCTGATGCACCTGCCCCCACCAC
TCACGCCGCCAACTCCTGTCCCCTGGGGTGGAATAAACGGGGCTCAGGTTTCAGAGGCCG
CAACCCCATCGCCCCTCCCAGCACAGAGACTAGAGGTCCCGGCCAACGGTGGGCCTCACG
GAGGGGCGACGACGCTCACCTCTCCTCTGCAGGATCCCGCGGTACGCCACCTCGTAGTTG
TGTCTGCACACCCTGTCCACCGACGCCCGGGCCCCCTCCAGGACTTCCTTCTGGCTGTTC
C
```

hifiasm-GIAB-TR haplotype 0

```
CTGCTTGTCTCCCTGCTCTGCCCTAGGTCCCCGCCCATCTGATGCACCTGCCCCACCACT
CACGCCGCCAACTCCTGTCCCCTGGGGTGGAATAAACGGGGCTCAGGTTTCAGAGGCCGC
AACCCCATCGCCCCTCCCAGCACAGAGACTAGAGGTCCCGGCCAACGGTGGGCCTCACGG
AGGGGCGACGACGCTCACCTCTCCTCTGCAGGATCCCGCGGTACGCCACCTCGTAGTTGT
GTCTGCACACCCTGTCCACCGACGCCCGGGCCCCCTCCAGGACTTCCTTCTGGCTGTTCC
```

Fig. S4: Full haplotype sequences for the truth (Q100-dipcall) and query (hifiasm-dipcall, Q100-PAV, hifiasm-GIAB-TR) VCFs for a portion of the HLA-DQB1 gene (`chr6:32,664,600-32,664,899`), with all variants applied to the reference sequence. Substitutions (SNPs) are highlighted in blue, insertions in green, and deletions in red. Please note that the first and second pairs of sequences are identical, despite differing variant representations.

Validation and Detailed Comparisons

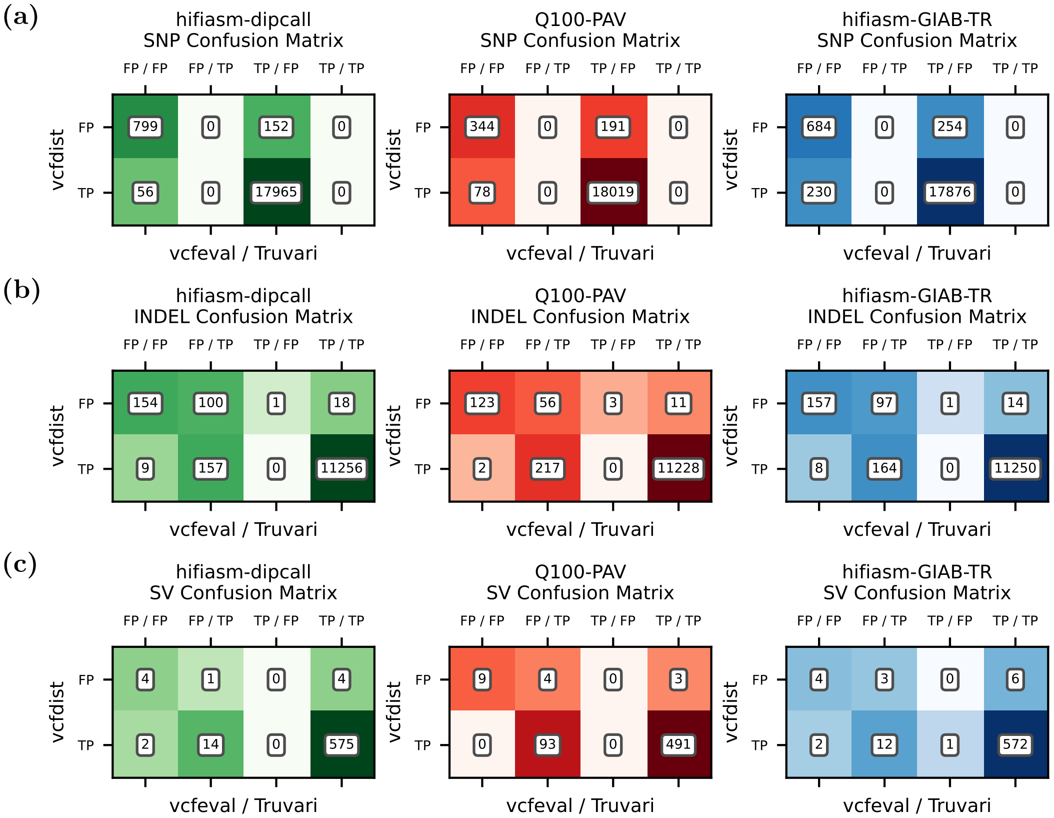

Fig. S5: A comparison of **(a)** SNP, **(b)** INDEL, and **(c)** SV variant calling evaluation by vcfdist, vcfeval, and Truvari, restricting to chr20 of the GIAB-TR tandem repeats BED. Prior to evaluation, truth and query VCFs were normalized using Truvari phab. This means that the Truvari results reported in this figure are largely equivalent to Truvari refine (MAFFT).

<table>
<tr><th></th><th>POS</th><th>REF</th><th>ALT</th><th>FORMAT</th><th>VCFEVAL TRUTH</th><th>VCFEVAL QUERY</th><th>TRUVARI TRUTH</th><th>TRUVARI QUERY</th><th>VCFDIST TRUTH</th><th>VCFDIST QUERY</th></tr>
<tr><td rowspan="2">Allele Match</td><td>40153469</td><td>GTATA</td><td>G</td><td>GT:BD:BK</td><td>0|1:FN:am</td><td>1|1:FP:am</td><td>0|1:TP:am</td><td>1|1:TP:am</td><td>0|1:TP:gm</td><td>0|1:TP:gm</td></tr>
<tr><td>40153469</td><td>GTATA</td><td>G</td><td>GT:BD:BK</td><td>.:.:.</td><td>.:.:.</td><td>.:.:.</td><td>.:.:.</td><td>.:.:.</td><td>1|0:FP:.</td></tr>
<tr><td rowspan="2">Different Thresholds</td><td>39864946</td><td>G</td><td>GTTT</td><td>GT:BD:BC</td><td>0|1:FN:0.0</td><td>.:.:.</td><td>0|1:TP:.75</td><td>.:.:.</td><td>0|1:FN:.67</td><td>.:.:.</td></tr>
<tr><td>39864946</td><td>G</td><td>GTTTT</td><td>GT:BD:BC</td><td>.:.:.</td><td>0|1:FP:0.0</td><td>.:.:.</td><td>0|1:TP:.75</td><td>.:.:.</td><td>0|1:FP:0.0</td></tr>
<tr><td rowspan="2">Complex Variant</td><td>11753707</td><td>C</td><td>CGTGTGTGTGT</td><td>GT:BD:BC</td><td>1|0:FN:0.0</td><td>.:.:.</td><td>1|0:FN:0.0</td><td>.:.:.</td><td>1|0:FN:.38</td><td>.:.:.</td></tr>
<tr><td>11753744</td><td>G</td><td>GTGTGTA</td><td>GT:BD:BC</td><td>1|0:TP:1.0</td><td>1|0:TP:1.0</td><td>1|0:TP:1.0</td><td>1|0:TP:1.0</td><td>1|0:FN:.38</td><td>1|0:FP:0.0</td></tr>
<tr><td rowspan="3">Pick Single</td><td>18945947</td><td>C</td><td>CAGAGAGAGAGAG</td><td>GT:BD:BC</td><td>.:.:.</td><td>1|0:FP:0.0</td><td>.:.:.</td><td>1|0:FP:0.0</td><td>.:.:.</td><td>1|0:TP:.80</td></tr>
<tr><td>18945947</td><td>C</td><td>CAGAGAGAGAG</td><td>GT:BD:BC</td><td>1|1:FN:0.0</td><td>0|1:FP:0.0</td><td>1|1:TP:1.0</td><td>0|1:TP:1.0</td><td>0|1:TP:1.0</td><td>0|1:TP:1.0</td></tr>
<tr><td>18945947</td><td>C</td><td>CAGAGAGAGAG</td><td>GT:BD:BC</td><td>.:.:.</td><td>.:.:.</td><td>.:.:.</td><td>.:.:.</td><td>1|0:TP:.80</td><td>.:.:.</td></tr>
<tr><td rowspan="3">Flip Error</td><td>33052546</td><td>G</td><td>GTA</td><td>GT:BD:BC</td><td>1|0:TP:1.0</td><td>1|0:FP:0.0</td><td>1|0:TP:1.0</td><td>1|0:TP:1.0</td><td>1|0:TP:1.0</td><td>1|0:TP:1.0</td></tr>
<tr><td>33052546</td><td>GTG</td><td>G</td><td>GT:BD:BC</td><td>0|1:TP:1.0</td><td>.:.:.</td><td>0|1:FN:0.0</td><td>.:.:.</td><td>0|1:FN:0.0</td><td>.:.:.</td></tr>
<tr><td>33052548</td><td>G</td><td>A</td><td>GT:BD:BC</td><td>.:.:.</td><td>0|1:TP:1.0</td><td>.:.:.</td><td>.:.:.</td><td>.:.:.</td><td>0|1:FP:0.0</td></tr>
<tr><td rowspan="2">Backtracking Tie</td><td>23651946</td><td>G</td><td>GC</td><td>GT:BD:BC</td><td>0|1:TP:1.0</td><td>0|1:TP:1.0</td><td>0|1:TP:1.0</td><td>0|1:TP:1.0</td><td>0|1:TP:1.0</td><td>0|1:FP:0.0</td></tr>
<tr><td>23651955</td><td>C</td><td>CC</td><td>GT:BD:BC</td><td>.:.:.</td><td>0|1:FP:0.0</td><td>.:.:.</td><td>0|1:FP:0.0</td><td>.:.:.</td><td>0|1:TP:1.0</td></tr>
<tr><td rowspan="3">Variant Overlap</td><td>35755334</td><td>C</td><td>CAT</td><td>GT:BD:BC</td><td>0|1:FN:0.0</td><td>0|1:TP:1.0</td><td>0|1:TP:1.0</td><td>0|1:TP:1.0</td><td>0|1:TP:1.0</td><td>0|1:TP:1.0</td></tr>
<tr><td>35755334</td><td>C</td><td>CATAT</td><td>GT:BD:BC</td><td>.:.:.</td><td>0|1:TP:1.0</td><td>.:.:.</td><td>0|1:FP:0.0</td><td>.:.:.</td><td>0|1:FP:0.0</td></tr>
<tr><td>35755334</td><td>C</td><td>CATATAT</td><td>GT:BD:BC</td><td>0|1:TP:1.0</td><td>.:.:.</td><td>0|1:FN:.67</td><td>.:.:.</td><td>0|1:FN:.67</td><td>.:.:.</td></tr>
</table>

Fig. S6: A simple real example for each of the seven categories of variants in Table 4 that were evaluated differently between tools. Each variant call file (VCF) entry shows the variant position (POS), reference and alternate alleles (REF and ALT), and supplementary information such as genotype (GT), benchmarking decision (BD), benchmarking category (BK, which is either a genotype (gm) or allele (am) match), and benchmarking credit (BC).

Clustering

For alignment-based variant calling evaluation tools it is necessary to group variants together into many independent clusters that can be evaluated individually, since aligning entire chromosomes is currently computationally infeasible. In order to understand the impact of variant clustering on the measured accuracy and runtime of evaluation tools, we explore and compare several different options for variant clustering. Truvari clusters variants by BED region during evaluation. vcfeval does not cluster variants since it is not alignment-based. Requiring exact variant matches allows it to use a branch-and-bound approach instead. For vcfdist, we implemented two options: a simple yet flexible variant clustering heuristic, and a more sophisticated clustering algorithm that attempts to minimize computation while retaining perfect accuracy.

The simplest option that vcfdist offers is to group all variants less than n bases apart into a single cluster. We call this "gap n" clustering, as shown in Additional File 1: Table S2. The average region size, runtime, and measured variant calling performance all depend highly upon n. Using a gap of $n = 10$ results in the fastest runtime, but at the cost of significantly lower measured SNP, INDEL, and SV accuracy. A larger gap of 100 is a reasonable balance between performance and accuracy, and only SV accuracy is noticeably impacted. A much larger gap of 500 almost completely eliminates cases where true positive variants are mistakenly labelled false positives due to equivalent query and truth variants not being clustered together, but at the cost of a much higher runtime.

We develop our own clustering method based on bidirectional wavefront alignment (biWFA) that dynamically clusters variants based on whether truth and query variants have the potential to be equivalent. It aligns leftwards and rightwards from each variant cluster, determining the span of reference bases that can be influenced by the current cluster of variants. This algorithm is able to find long-range variant dependencies that may span repetitive regions of the genome longer than 500bp, while also splitting up independent variants that are nearby on the reference. As shown in Additional File 1: Table S2, this approach achieves even higher accuracy than gap 500 clustering, whilst running more than 10× faster end-to-end.

Clustering approach	None	GIAB-Q100 BED	Gap 10	Gap 100	Gap 500	BiWFA
**Total regions**	24	300	**4,363,640**	3,343,352	1,503,147	4,259,395
**Total regions size**	3,088,269,832	2,789,029,111	**18,548,559**	69,515,796	550,160,873	24,589,303
**Mean region size**	128,677,910	9,296,764	**4.25**	20.8	366	5.77
**Max region size**	248,956,422	127,461,328	**2,522**	19,730	65,503	29,663
**SNP F1 score**	*	*	0.9644	0.9719	0.9722	**0.9723**
**INDEL F1 score**	*	*	0.9415	0.9626	**0.9631**	**0.9631**
**SV F1 score**	*	*	0.8244	0.9427	0.9514	**0.9516**
**Runtime**	*	*	**00:02:57**	00:11:53	11:25:06	01:04:56

Table S2: The efficiency and measured accuracy of evaluating the Q100-PAV VCF with vcfdist on the Q100-dipcall ground truth VCF using different clustering approaches. Accuracy entries marked with * were unable to be computed due to long runtimes caused by large clustered regions. Clustering variants together whenever they are less than 10 bases apart ("Gap 10") is the most efficient method, although its accuracy is significantly reduced. Bidirectional wavefront alignment (biWFA) based clustering achieves the highest accuracy with reasonable performance.

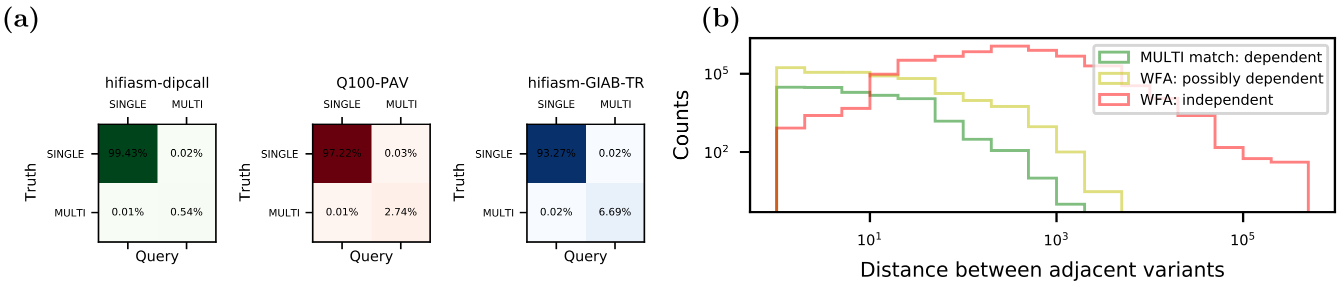

Fig. S7: (a) As variant representation becomes increasingly different from the Q100-dipcall ground truth (shown in Additional File 1: Fig. S1), the fraction of variants requiring complex comparisons involving multiple truth and query variants increases. **(b)** vcfdist's biWFA clustering algorithm groups possibly dependent variants (yellow) together, and starts a new cluster when two adjacent variants are independent (red). Of the variants clustered together, only a small fraction are actually dependent (green). However, grouping these variants together is necessary to correctly determine equivalence between sets of truth and query variants.

Query VCF			Truth VCF		
POS	REF	ALT	POS	REF	ALT
2	A	G	2	A	G
4	A	AC	6	C	CCT
6	C	CT	15	T	C
10	A	ACA	16	A	ATA
18	CGC	C	18	CGCT	C
23	T	A	23	T	TA

Legend

Query	Truth
SINGLE	SINGLE
SINGLE	MULTI
MULTI	SINGLE
MULTI	MULTI

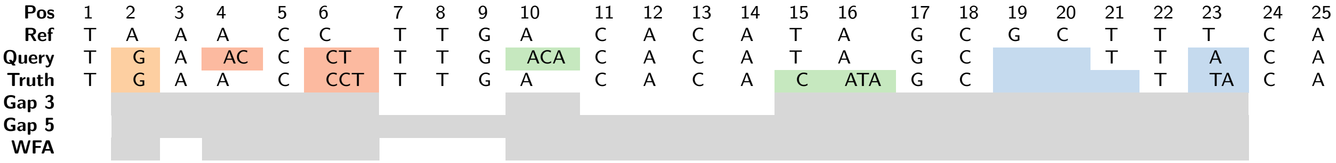

Fig. S8: An example of different variant clustering methods. The span of each cluster is shown in gray. Equivalent sets of truth and query variants are depicted in different colors, and one example from each category is included. The four categories describe whether a single or multiple truth or query variants participate in the match. For vcfdist to determine variant equivalence, all dependent variants must be located in the same cluster. Incorrectly separating variants into different clusters results in lower measured accuracy. Unnecessarily grouping variants together results in larger clusters and more expensive evaluation. This tradeoff is shown in Additional File 1: Table S2.
